# Supplementary material for: Canadian Guideline on the Management of a Positive Human Papillomavirus Test and Guidance for Specific Populations
Source: Curr Oncol. 2023 Jun 9;30(6):5652–79. doi: 10.3390/curroncol30060425 (PMC10297596; doi:10.3390/curroncol30060425)
Supplement: Supplementary file 1 [file curroncol-30-00425-s001.zip › Supplementary Table S1.pdf]

Table S1. Literature search strategy—Search for existing clinical practice guidelines related to HPV screening.

MSSU USSM

Requestor:	James Bentley (via Tiffany Zigras)
Request Date:	7 June 2021
Project:	Management of a HPV Positive Test
Research Question:	Search for existing clinical practice guidelines related to HPV screening
Target Articles:	Clinical practice guidelines

Search Histories

Database: Ovid MEDLINE All
Date searched: 7 June 2021

#	Searches	Results
1	exp clinical pathway/	7392
2	exp clinical protocol/	182538
3	exp consensus/	17768
4	exp consensus development conference/	12573
5	exp consensus development conferences as topic/	2990
6	critical pathways/	7392
7	exp guideline/	36834
8	guidelines as topic/	41941
9	exp practice guideline/	29652
10	practice guidelines as topic/	126962
11	health planning guidelines/	4157
12	(guideline or practice guideline or consensus development conference or consensus development conference, NIH).pt.	46651
13	(position statement* or policy statement* or practice parameter* or best practice*).ti,ab,kf,kw.	40446
14	(standards or guideline or guidelines).ti,kf,kw.	124632
15	((practice or treatment* or clinical) adj guideline*).ab.	47060
16	(CPG or CPGs).ti.	6144
17	consensus*.ti,kf,kw.	30978
18	consensus*.ab. /freq=2	30076
19	((critical or clinical or practice) adj2 (path or paths or pathway or pathways or protocol*)).ti,ab,kf,kw.	23820

20	recommendat*.ti,kf,kw.	48222
21	(care adj2 (standard or path or paths or pathway or pathways or map or maps or plan or plans)).ti,ab,kf,kw.	72362
22	(algorithm* adj2 (screening or examination or test or tested or testing or assessment* or diagnosis or diagnoses or diagnosed or diagnosing)).ti,ab,kf,kw.	9145
23	(algorithm* adj2 (pharmacotherap* or chemotherap* or chemotreatment* or therap* or treatment* or intervention*)).ti,ab,kf,kw.	11615
24	or/1-23 [CADTH CPG filter]	693013
25	exp Alphapapillomavirus/	9034
26	Betapapillomavirus/	151
27	Gammapapillomavirus/	67
28	Mupapillomavirus/	9
29	or/25-28	9194
30	exp Early Diagnosis/	61270
31	(test* or screen* or detect* or diagnos* or self sampl*).ti,kw,kf.	1799612
32	(genotyp* or methylat*).ti,ab,kw,kf.	472935
33	(ki67* or p16*).ti,ab,kw,kf.	32630
34	or/30-33	2292971
35	29 and 34	3482
36	(human papillomavirus or hpv* or alphapapillomavirus or betapapillomavirus or gammapapillomavirus or mupapillomavirus).ti.	34901
37	30 and 36	1639
38	((human papillomavirus or hpv* or alphapapillomavirus or betapapillomavirus or gammapapillomavirus or mupapillomavirus) adj5 (test* or screen* or detect* or self sampl* or genotyp* or methylat* or ki67* or p16*)).ti,ab,kw,kf.	20581
39	Human Papillomavirus DNA Tests/	566
40	DNA Probes, HPV/	1070
41	or/38-40	21036
42	35 or 37 or 41	21698
43	24 and 42	913
