# Supplementary material for: Canadian Guideline on the Management of a Positive Human Papillomavirus Test and Guidance for Specific Populations
Source: Curr Oncol. 2023 Jun 9;30(6):5652–79. doi: 10.3390/curroncol30060425 (PMC10297596; doi:10.3390/curroncol30060425)
Supplement: Supplementary file 1 [file curroncol-30-00425-s001.zip › Supplementary Table S2.pdf]

Table S2. Literature Search Strategy—HPV testing for cervical screening in specific contexts and populations.

Requestor:	James Bentley (via Tiffany Zigras)
Request Date:	14 October 2021
Project:	HPV testing CPG
Research Question:	5 questions below (2018-present)
Target Articles:	

1. How to triage a positive HPV test
2. Self-sampling HPV test (e.g. acceptability, reliability, use in non-attenders)
3. HPV test results in the immunocompromised population
4. HPV test results in the LGBTQ+ population
5. HPV tests and rural/first nations/newcomer populations (i.e. under screened)

Search Histories

10 November 2021

Ovid Medline

Search saved as Bentley_HPVTesting_2021-11-10

Ovid MEDLINE(R) ALL <1946 to November 09, 2021>

#	Search terms	Results	Notes
1	exp Alphapapillomavirus/	8638	
2	Betapapillomavirus/	150	
3	Gammapapillomavirus/	66	
4	Mupapillomavirus/	9	
5	exp Papillomavirus Infections/	37992	
6	(human papillomavirus or hpv* or alphapapillomavirus or betapapillomavirus or gammapapillomavirus or mupapillomavirus).ti,ab,kw,kf.	55780	
7	or/1-6	66192	
8	exp Early Diagnosis/	59526	
9	(test* or screen* or detect* or diagnos* or self sampl*).ti,ab,kw,kf.	7862223	
10	(genotyp* or methylat*).ti,ab,kw,kf.	464635	
11	(ki67* or p16*).ti,ab,kw,kf.	31970	

12	or/8-11	8153586	
13	Human Papillomavirus DNA Tests/	557	
14	DNA Probes, HPV/	1070	
15	or/13-14	1613	
16	7 and 12	35721	
17	15 or 16	35983	
18	(triag* or manage* or progress*).ti,ab,kw,kf.	2651099	
19	((human papillomavirus or hpv*) adj2 positiv*).ti,ab,kw,kf.	10435	
20	18 and 19	2266	
21	limit 20 to yr="2018 -Current"	750	Question 1 results
22	self sampl*.ti,ab,kw,kf.	881	
23	non attender*.ti,ab,kw,kf.	497	
24	7 and 22 and 23	21	
25	limit 24 to yr="2018 -Current"	13	Question 2 results
26	exp Immunocompromised Host/	26632	
27	(Immunocompromise* or immunosuppress* or immunodeficien*).ti,ab,kw,kf.	337755	
28	or/26-27	350183	
29	17 and 28	1929	
30	limit 29 to yr="2018 -Current"	466	Question 3 results
31	exp "Sexual and Gender Minorities"/	10669	
32	bisexuality/ or exp homosexuality/ or transsexualism/	35983	
33	(homosexual* or gay or gays or lesbian* or LGB* or bisexual* or queer or "sexual and gender minorit*" or sexual minorit* or men who have sex with men or women who have sex with women or two-spirit* or gender minorit* or genderqueer or intersex* or transgender* or trans female or trans male or transman or trans man or transmen or trans men or transpeople or trans people or transwoman or trans woman or transwomen or trans women or transsexual*).ti,ab,kw,kf.	53330	
34	or/31-33	63057	
35	17 and 34	871	
36	limit 35 to yr="2018 -Current"	300	Question 4 results
37	exp Indigenous Canadians/	4069	
38	Rural Population/	64970	
39	Rural Health/	23744	
40	exp "Emigrants and Immigrants"/	14171	
41	Refugees/	11628	
42	(Indigenous or first nation* or metis or inuit or aboriginal or native or rural or immigrant* or newcomer* or refugee*).ti,ab,kw,kf.	463528	
43	or/37-42	494039	
44	17 and 43	923	
45	limit 44 to yr="2018 -Current"	316	Question 5 results
