# Supplementary material for: Canadian Guideline on the Management of a Positive Human Papillomavirus Test and Guidance for Specific Populations
Source: Curr Oncol. 2023 Jun 9;30(6):5652–79. doi: 10.3390/curroncol30060425 (PMC10297596; doi:10.3390/curroncol30060425)
Supplement: Supplementary file 1 [file curroncol-30-00425-s001.zip › Supplementary Table S3.pdf]

Table S3. Literature Search Strategy—How to manage a positive HPV test after a hysterectomy.

Requestor:	James Bentley (via Tiffany Zigras)
Request Date:	4 February 2022
Project:	HPV testing CPG
Research Question:	How to manage a positive HPV test after a hysterectomy (2018-present)
Target Articles:	

Search Histories

7 February 2022

Ovid Medline

Search saved as Bentley_HPVTesting_Hysterectomy_2022-02-07

Ovid MEDLINE(R) ALL <1946 to February 04, 2022>

#	Search terms	Results
1	exp Alphapapillomavirus/	8972
2	Betapapillomavirus/	151
3	Gammapapillomavirus/	67
4	Mupapillomavirus/	9
5	exp Papillomavirus Infections/	38703
6	(human papillomavirus or hpv* or alphapapillomavirus or betapapillomavirus or gammapapillomavirus or mupapillomavirus).ti,ab,kw,kf.	56627
7	or/1-6	67106
8	exp Early Diagnosis/	61027
9	(test* or screen* or detect* or diagnos* or self sampl*).ti,ab,kw,kf.	7981897
10	(genotyp* or methylat*).ti,ab,kw,kf.	471508
11	(ki67* or p16*).ti,ab,kw,kf.	32520
12	or/8-11	8277312
13	Human Papillomavirus DNA Tests/	564
14	DNA Probes, HPV/	1070
15	or/13-14	1620
16	7 and 12	36269
17	15 or 16	36531
18	exp Hysterectomy/	32591
19	Hysterectom*.ti,ab,kw,kf.	40494
20	or/18-19	51843
21	17 and 20	447
22	limit 21 to yr="2018 -Current"	123
