# Supplementary material for: Canadian Guideline on the Management of a Positive Human Papillomavirus Test and Guidance for Specific Populations
Source: Curr Oncol. 2023 Jun 9;30(6):5652–79. doi: 10.3390/curroncol30060425 (PMC10297596; doi:10.3390/curroncol30060425)
Supplement: Supplementary file 1 [file curroncol-30-00425-s001.zip › Supplementary Table S4.pdf]

Table S4. Grade of Recommendations and Evaluation of Quality.

**1: Strength and Quality of Evidence Grades**	
Grade	Definition
Strength of Recommendation	
Strong	High level of confidence that the desirable effects outweigh the undesirable effects (strong recommendation for) or the undesirable effects outweigh the desirable effects (strong recommendation against)
Conditional	Desirable effects probably outweigh the undesirable effects (weak recommendation for) or the undesirable effects probably outweigh the desirable effects (weak recommendation against)
**Quality of evidence**	
High	We are very confident that the true effect lies close to that of the estimate of the effect.
Moderate	We are moderately confident in the effect estimate: The true effect is likely to be close to the estimate of the effect, but there is a possibility that it is substantially different.
Low	Our confidence in the effect estimate is limited: The true effect may be substantially different from the estimate of the effect.
Very Low	We have very little confidence in the effect estimate: The true effect is likely to be substantially different from the estimate of effect.

Adapted from GRADE handbook table 5.1

2: Implications of strong and weak recommendations for different users of guidelines		
	Strong Recommendation	Weak Recommendation
**For patients**	Most individuals in this situation would want the recommended course of action and only a small proportion would not.	The majority of individuals in this situation would want the suggested course of action, but many would not.
**For clinicians**	Most individuals should receive the recommended course of action. Adherence to this recommendation according to the guideline could be used as a quality criterion or performance indicator. Formal decision aids are not likely to be needed to help individuals make decisions consistent with their values and preferences.	Recognize that different choices will be appropriate for different patients, and that you must help each patient arrive at a management decision consistent with her or his values and preferences. Decision aids may well be useful helping individuals making decisions consistent with their values and preferences. Clinicians should expect to spend more time with patients when working towards a decision.
**For policy makers**	The recommendation can be adapted as policy in most situations including for the use as performance indicators.	Policy making will require substantial debates and involvement of many stakeholders. Policies are also more likely to vary between regions. Performance indicators would have to focus on the fact that adequate deliberation about the management options has taken place.

Adapted from GRADE handbook table 6.1

Grade Handbook available at: https://gdt.gradepro.org/app/handbook/handbook.html
